# Heat shock protein 70 expression induced by diode laser irradiation on choroid-retinal endothelial cells in vitro

**Published:** 2012-09-21

**Authors:** Shanshan Du, Qiong Zhang, Shisheng Zhang, Ling Wang, Jingcai Lian

**Affiliations:** Department of Ophthalmology, Ruijin Hospital, School of Medicine, Shanghai Jiaotong University, Shanghai, China

## Abstract

**Purpose:**

To investigate the biologic effect of an 810 nm diode laser on the induction of heat shock protein 70 (Hsp70) in choroid-retinal endothelial cells in vitro.

**Methods:**

Cultured rhesus macaque choroid-retinal endothelial cells (RF/6A) were irradiated using an 810 nm diode laser (spot size, 10 mm; duration, 60 s; power, 400–1,500 mW). Cell viability was assessed by annexin V- fluorescein isothiocyanate (FITC) and propidium iodide flow cytometric assay. Hsp70 expression was determined by western blot at 6, 12, 18, 24, and 48 h following laser exposure. Intracellular distribution of Hsp70 was examined by immunofluorescence staining.

**Results:**

The laser-induced cell injury threshold was found to be at a power of 1,100 mW power (fluence, 84.08 J/cm^2^), above which there was significant cell death. Under this power, Hsp70 expression elevated obviously and was stronger at 600–1,000 mW power settings (fluences, 45.86–76.43 J/cm^2^). The expression of Hsp70 peaked at 12–18 h postirradiation, and returned to baseline by 48 h. Immunofluorescence staining indicated the induced Hsp70 expression in both the cytoplasm and the nucleus.

**Conclusions:**

Subthreshold 810 nm diode laser exposure can induce Hsp70 hyperexpression from 12 to 18 h postirradiation in cultured choroid-retinal endothelial cells without obvious cell death. The results could be useful for investigating and designing more effective laser therapies.

## Introduction

Choroidal neovascularization (CNV) causes severe vision loss in several eye disorders such as age-related macular degeneration, ocular histoplasmosis, and high myopia [1]. Minimally invasive laser therapies have been commonly used, such as laser photocoagulation, photodynamic therapy (PDT), and transpupillary thermotherapy (TTT). Conventional laser photocoagulation is a well established treatment for some forms of CNV; nevertheless, it frequently damages the collateral neural retina, resulting in irreversible effects, including scotomata and the disruption of the retinal structure through scarring. PDT is an approach that specifically occludes subfoveal CNV without significant damage to adjacent tissues; however, a high proportion of secondary CNV and persistent choriocapillaris hypoperfusion have been found to occur with PDT and eventually lead to poor visual prognosis [2,3].

TTT, which is a long-exposure, large spot size, low-irradiance, near-infrared (diode 810 nm) laser protocol, slowly increases tissue temperature to approximately 4–10 °C above basal levels [4]. This procedure has been recently used with some success to slow down the progression of exudation and block subfoveal CNV in age-related macular degeneration [5-7]. However, clinical use of TTT for CNV has been limited due to its lack of efficacy and occasional retinal damage [8,9]. It is difficult to devise an optimal laser parameter. Undertreatment would not close CNV, while overtreatment would lead to localized retinal destruction. Furthermore, the underlying mechanism of TTT remains unclear.

Heat shock proteins (Hsps) are a group of ubiquitous, highly conserved proteins in all organisms, and are classified according to their molecular mass. Elevated Hsps can be triggered by a variety of stressful stimuli and protect cells against stress. The 70 kDa Hsp (Hsp70) is particularly known to be induced by thermal, ischemic, and oxidative stress. Acting as a molecular chaperone, Hsp70 can assist in the refolding of denatured proteins and inhibit improper protein aggregation [10-12]. Moreover, the induction of Hsp70 may be responsible for the protective effect against apoptosis and inflammation [13-17]. A few animal experiments have found increased Hsp70 expression in the choroidoretinal layers [18-21] or optic nerve head [22] following TTT. From this perspective, TTT may be a subthreshold thermotherapy inducing Hsp70 hyperexpression, modulating apoptosis in choroidoretinal tissues [5,20,22,23]. Careful application of laser to induce Hsp70 hyperexpression may serve a protective role. Therefore, a better understanding of laser-induced Hsp70 kinetics is essential for the design of effective laser protocols. However, comprehensive Hsp70 expression kinetics data for choroidoretinal tissues or choroidoretinal related cells following TTT have not been reported.

In this study, we focus on Hsp70 expression induced by 810 nm diode laser exposure in cultured choroid-retinal endothelial cells. We aim to elaborate on the conditions in which the protein is hyperexpressed, as well as the threshold at which cellular damage occurs.

## Methods

### Cell culture

RF/6A rhesus macaque choroid-retinal endothelial cells were obtained from the Shanghai Institutes for Biologic Sciences (Shanghai, China) and were grown as monolayers in RPMI 1640 culture medium (Hyclone, Logan, UT) supplemented with 15% fetal bovine serum (Hyclone) plus 100 U/ml penicillin/streptomycin mixture (Sigma-Aldrich, Saint Louis, MO) in a 5% CO_2_ humidified atmosphere at 37 °C. Prior to laser irradiation, cells were seeded in 24-well culture plates (4×10^4^ cells/well) to grow to reach about 80% confluence (about 3 days).

### Laser irradiation

**Figure 1:**
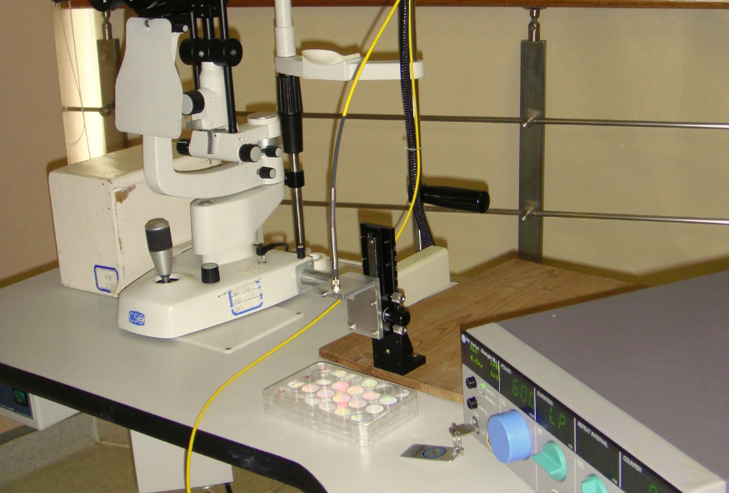
The setting for laser irradiation on RF/6A cells. Laser light was delivered through a 200 µm fiber-optic system, and the distance between the laser source and the cell monolayer was adjusted to obtain a laser spot that was 10 mm in diameter.

Irradiation was performed using an 810 nm diode laser (OcuLight SLx; Iridex Corporation, Mountain View, CA), delivered through a 200 µm fiber-optic system with different power outputs for 60 s in continuous wave mode. The distance between the laser source and the cell monolayer was adjusted to achieve a 10 mm diameter laser spot (Figure 1), adequate to cover about half a well of a 24-well plate. Before laser exposure, the medium was removed, the cells were washed once in sterile phosphate buffered saline (PBS; 137 mM NaCl, 5.4 mM KCl, 1.28 mM NaH_2_PO_4_, 7 mM Na_2_HPO_4_; pH 7.4) and then transferred to no phenol red RPMI 1640 medium (Gibco, Grand Island, NY) without fetal bovine serum. The no phenol red medium was used to cover the cells during exposure to avoid absorption of laser energy by the culture medium. Following irradiation, fresh culture medium was added and the cells were incubated for an additional period for measurements. The control group was treated in the same manner, except the laser irradiation. Another group without any treatment was defined as the normal group.

First, we observed the morphological change of RF/6A cells after laser exposure under an inverted microscope (Nikon TE2000-U; Nikon, Tokyo, Japan), and then identified the power required to cause significant cell death by annexin V-fluorescein isothiocyanate (annexin V-FITC) and propidium iodide (PI) flow cytometric assay (threshold application). Using a spot diameter of 10 mm and a power setting of 1,100 mW or greater, 60 s of laser irradiation produced significant cell death. Then, the induced Hsp70 expression was studied at various subthreshold power settings, using decrements of 100 mW (1,000, 900, 800, … and 400 mW).

### Detection of cell viability

Flow cytometry using Annexin V-FITC and PI double staining (BD Biosciences, San Diego, CA) was employed to detect cell viability. Following 6 h of incubation after exposure, cells were washed with PBS and resuspended in 60 µl binding buffer. annexin V-FITC (3.8 µl) and 3.8 µl PI were sequentially added to the cell solution. After incubation for 10 min in the dark, the stained cell solution was diluted with another 120 µl binding buffer and directly analyzed using the FACSCalibur system (BD Biosciences).

### Western blot analysis

Based on the previous in vivo experimental data [18-20], we first detected Hsp70 expression with different subthreshold powers at 24 h postirradiation. Afterwards, the power resulting in stronger Hsp70 expression was used for observation of Hsp70 levels at 6, 12, 18, 24, and 48 h after irradiation. The cells were washed with ice-cold PBS and then lysed with radio immunoprecipitation assay (RIPA) buffer (Shenneng Bocai Biotechnology Co. Ltd, Shanghai, China) containing 1 mM phenylmethanesulfonyl fluoride (Shenneng Bocai Biotechnology Co. Ltd). The protein lysates were centrifuged at 12,000 × *g* at 4 °C for 30 min and the supernatant was collected for protein quantification using the bicinchoninic acid assay kit (Pierce, Rockford, IL). Equal amounts of protein (10–15 µg per lane) were separated by 10% sodium dodecyl sulfate PAGE and transferred onto a nitrocellulose membrane (Whatman Protran, Cambridge, UK). After blocking with 5% skimmed milk in 10 mmol/l Tris-buffered saline with 0.1% Tween-20 for 1 h, the blotted membrane was incubated with mouse monoclonal anti-Hsp70 antibody (1:1,000; Abcam, Cambridge, UK, no cross-reactivity to Hsc70) overnight at 4 °C. After washing three times with 10 mmol/l Tris-buffered saline with 0.1% Tween-20, the membrane was incubated with IRDye 800CW goat anti-mouse secondary antibody (LI-COR, Lincoln, NE) for 1 h at room temperature. The signals were detected using an Odyssey Infrared Imaging System (LI-COR). Band intensity was analyzed by Quantity One software and compared with the internal standard glyceraldehyde-3-phosphate dehydrogenase (1:1,000; Abcam).

### Immunofluorescence assay

Cells were prepared in triplicate on 0.1% gelatin-coated glass coverslips and treated with the laser, as described above. At 18 h after irradiation, adherent cells were fixed in 4% paraformaldehyde for 15 min, blocked with 10% goat serum containing 0.5% Triton X-100 in PBS for 30 min, and then incubated with mouse monoclonal anti-Hsp70 antibody (1:100; Abcam) overnight at 4 °C. After three rinses with PBS, the samples were incubated with Cy3-conjugated goat anti-mouse secondary antibody (1:100, Jackson ImmunoResearch Laboratories, Inc., West Grove, PA) for 1 h at room temperature in the dark. The slides were then washed in PBS and stained with 300 nM diamidinophenylindole (Sigma-Aldrich, Saint Louis, MO) for 10 min followed by three rinses with PBS. After mounting with fluorescent mounting media (Dako, Glostrup, Denmark), the slides were observed under a fluorescence microscope (Nikon 80i; Nikon).

### Statistical analysis

All experiments were repeated at least three times. Statistical analysis was performed using SPSS 16.0 software. All error bars represent standard error of the mean (SEM). Differences between the laser-treated and control groups were evaluated by the Student paired *t* test. Statistical significance was defined as p<0.05.

## Results

### Inverted microscopy

**Figure 2:**
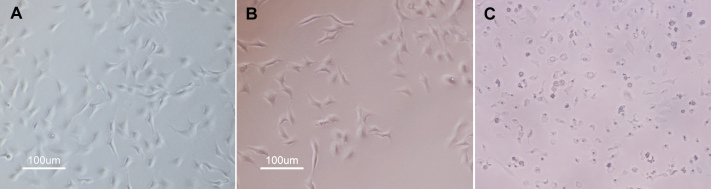
Micrographs of RF/6A cells treated with different laser powers. **A**: Unirradiated RF/6A cells. **B**: RF/6A cells treated with 1,000 mW laser irradiation. **C**: RF/6A cells treated with 1,100 mW laser irradiation. Cells with apoptotic or necrotic morphology were present in the 1,100 mW treated samples. Data are from one of three separate experiments with similar results.

The morphology of RF/6A cells treated with laser power lower than 1,100 mW was similar to that of the control (Figure 2A,B). However, a large number of cells with apoptotic or necrotic morphology (small, adherent or floating, rounded, or bubbling and shrunken) were present in samples treated with 1,100 mW laser power (Figure 2C).

### Cell viability detection

**Figure 3:**
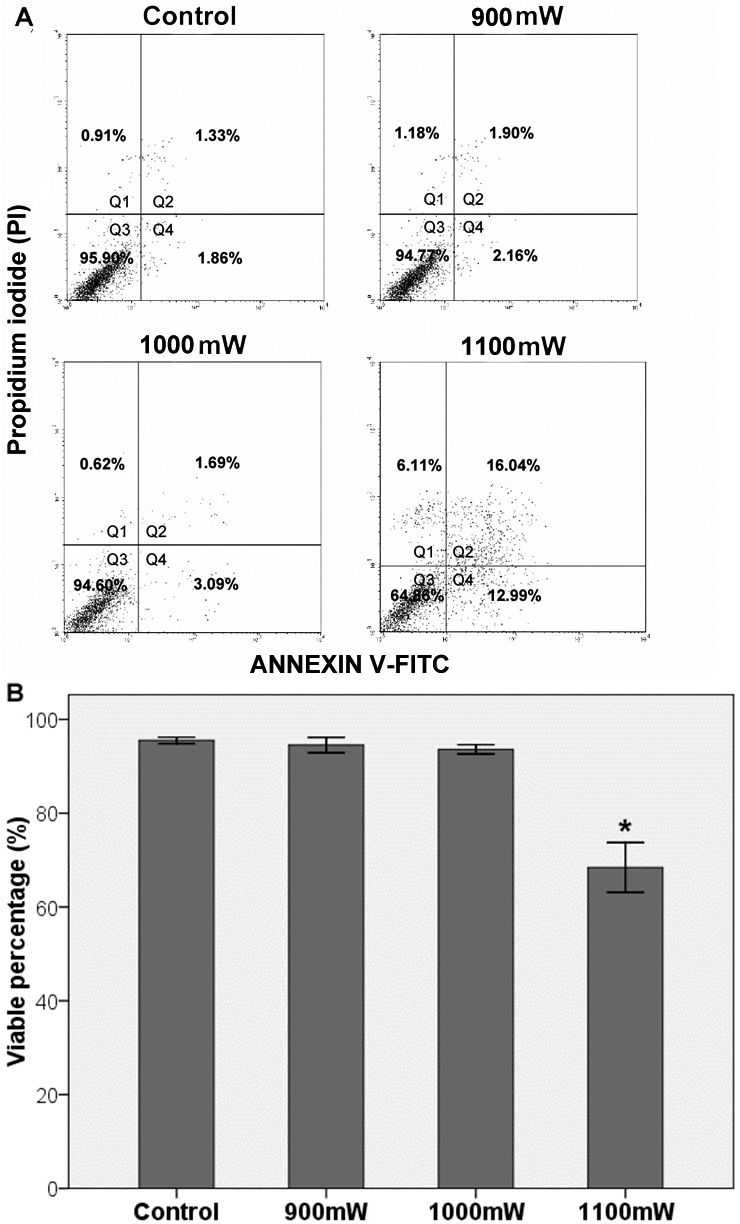
Flow cytometry analysis for the levels of cell viability 6 h following laser irradiation using different powers based on annexin V-fluorescein isothiocyanate and propidium iodide double staining. **A**: Representative flow cytogram. The percentage of cells in the Q3 region denotes viable cells, Q4: early apoptotic, Q1: necrotic, and Q2: late apoptotic/necrotic cells. **B**: Graphical quantitation of the cell viability rates. The data represent mean ± standard error of the mean (SEM) of three separate experiments (*p<0.05 versus control cells, Student *t* test). The results revealed that the laser injury threshold was at a laser power of 1,100 mW, above which there was significant cell death.

We performed a flow cytometry experiment of double staining with Annexin V-FITC and PI to determine the cell viability and the laser injury threshold. The cells were distinguished into the following four groups (Figure 3A): those that were unlabeled (viable cells), those that were stained with annexin V-FITC only (early apoptotic), those that were stained with PI only (necrotic), and those that were stained with both annexin V and PI (late apoptotic/necrotic cells). Necrotic cells and the cells in early- and late-stage apoptosis were defined as dead.

In the control group, the percentage of viable cells was 95.54%±0.34%. After laser treatment with 900 mW, 1,000 mW, or 1,100 mW power, the percentages of viable cells were 94.54%±0.82%, 93.63%±0.50%, and 68.44%±2.66%, respectively (Figure 3B). Significant difference was observed between the 1,100 mW and the control, 900 mW, and 1,000 mW groups (p=0.008, 0.009, and 0.013, respectively).

### Western blot analysis

**Figure 4:**
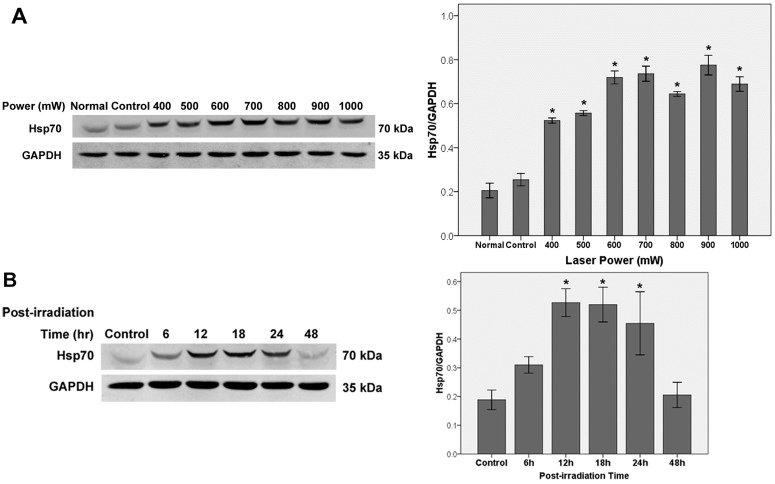
Hsp70 expression of RF/6A cells after laser irradiation. **A**: RF/6A cells treated with various laser powers and followed by 18 h incubation. **B**: RF/6A cells treated with 700 mW laser power and followed by various incubation times. Glyceraldehyde-3-phosphate dehydrogenase (GAPDH) was used as an internal control. Data are the mean±standard error of the mean (SEM) of three separate experiments (*p<0.05 versus control cells, Student *t* test).

Figure 4 shows the western blot results of Hsp70 expression for both a variable laser power with a fixed postirradiation period and a fixed laser power with a variable postirradiation period. The results demonstrated that Hsp70 was expressed at a very low level in unirradiated cells. After laser exposure with a lower power (400–1,000 mW) followed by 18 h recovery, the protein expression elevated to different levels and was stronger at 600–1,000 mW power settings (fluences, 45.86–76.43 J/cm^2^, Figure 4A).

We employed 700 mW (fluence, 53.50 J/cm^2^) laser power for observation of Hsp70 expression at different recovery times. Hsp70 expression at 1 and 3 h after laser exposure were similar to that of control (data not shown). The maximum expression of Hsp70 after laser exposure occurred at 12–18 h (about three times higher than the basal level), and gradually dropped to baseline at 48 h. Statistical analysis exhibited that Hsp70 expressions at 12, 18, and 24 h were all significantly higher than control (p<0.05, Figure 4B).

### Immunofluorescence assay

**Figure 5:**
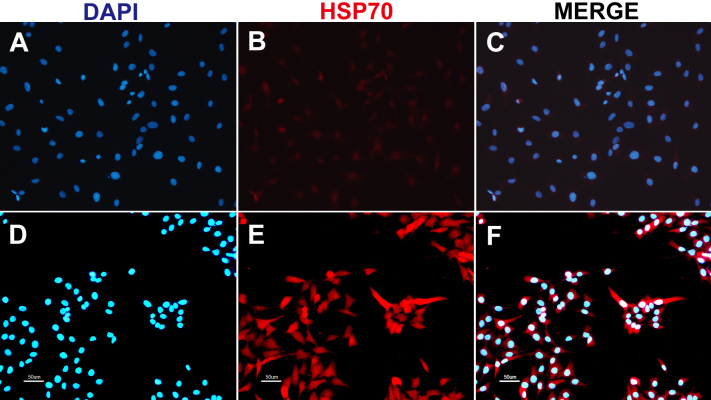
Expression and distribution of heat shock protein 70 in RF/6A cells after laser irradiation. **A**-**C**: Unirradiated RF/6A cells. **D**-**F**: RF/6A cells were treated with 700 mW laser irradiation and followed by 18 h incubation. Cells were fluorescently labeled with heat shock protein 70 (Hsp70; center, red channel) and the nuclear marker diamidinophenylindole (left, blue channel). The right is an overlay of the two channels. A strong induction of Hsp70 was observed in cells treated with a laser power of 700 mW. The induced Hsp70 was present in both the cytoplasm and nucleus. Data are from one of three separate experiments with similar results.

Immunofluorescence was performed to determine the expression and localization of induced Hsp70 in RF/6A cells. Cells were irradiated with 700 mW power, as already described, and incubated for 18 h. Unirradiated cells were used as control. As shown in Figure 5, Hsp70 was expressed at basal level in the nucleus and cytoplasm in unirradiated cells. After 700 mW laser exposure, a strong increase of Hsp70 was induced in both the cytoplasm and nucleus.

## Discussion

Laser irradiation at 810 nm, which was minimally absorbed by clear ocular media, was mostly delivered to retinal pigment epithelial and choroidal melanin. The absorption of the radiation could cause a limited temperature rise that may achieve occlusion of CNV [23,24]. It has recently been suggested that TTT could limit inflammation and play a major role in apoptosis and Hsp70 expression [23]. Hsp70 is necessary to make cells evolve toward survival instead of apoptosis or toward apoptosis instead of necrosis [25].

In effort to understand the laser-tissue interactions, some animal models have been investigated [18-21,26]. However, further biochemical and molecular analyses are not easily conducted through animal studies. Therefore, it has been difficult to devise an optimal therapeutic method based on a thorough understanding of the underlying mechanisms. In this study, we designed a laser-induced cell damage model to facilitate biologic research at the molecular level. To study the biologic effects on the cells, it is important to ensure that more cells are irradiated. Since the small spot diameters of commonly used lasers limit the number of cells irradiated, we selected a spot size of 10 mm in diameter to cover about half a well, so as to receive the most prominent cell responses.

In animal models, the laser power that induced retinal whitening was thought to be the photocoagulation threshold [19-21]. In our in vitro experiment, the laser-induced cell injury threshold was determined by annexin V-FITC and PI flow cytometric assay. Significant cell death was found at a laser power of 1,100 mW (fluence, 84.08 J/cm^2^). Moreover, the results showed that 1,100 mW power induced about 31.56%±2.66% cell death. As the laser spot in our experiment only covered half a well, the actual percentage of 1,100 mW laser induced cell death could be more than 50%. In addition, the inverted microscopy showed a large number of apoptotic or necrotic cells in samples treated with 1,100 mW power. Therefore, we assumed that this laser power of 1,100 mW (fluence, 84.08 J/cm^2^) corresponded to the laser injury threshold.

After the cellular damage threshold was identified, a range of subthreshold powers from 400 to 1,000 mW was applied for detection of Hsp70 expression (with fluences ranging from 30.57 to 76.43 J/cm^2^). With these parameters, Hsp70 expression was highly induced, especially at 600–1,000 mW power settings (fluences, 45.86–76.43 J/cm^2^). As indicated by immunofluorescence staining, induced expression of Hsp70 was present in both the cytoplasm and nucleus. Although the exposure conditions for Hsp70 induction in cultured cells in our study differed from those observed in vivo, their proportions of fluences for Hsp70 hyperexpression to fluences for the injury threshold are similar, about 60%–80% [19,20,26].

Hsp70 expression in our study was tracked for 48 h following laser exposure. The results showed that peak Hsp70 expression occurred at 12–18 h postirradiation, and reverted to baseline at 48 h, similar to the observations of Wang et al. [27], who used a matrix of heating and recovery times to determine the Hsp70 expression kinetics of bovine aortic endothelial cells in response to thermal stress. Kinetics data of Hsp70 expression following TTT in vivo have rarely been reported. In animal models, Hsp70 expression was mainly studied at 24 h after TTT.

The mechanisms of Hsp70 induction following 810 nm laser irradiation and the biologic action of Hsp70 merit further investigation. Since our RF/6A cells contained little melanin, they absorbed minimally with laser exposure, indicating that the induction of Hsp70 after laser exposure may be due to some nonthermal effects.

There were some limitations in our study. The laser spots were large in diameter and performed one by one, which may have led to nonuniform energy distribution and a nonuniform cellular response. Raster scanning may produce homogeneous exposure of cells and yield uniform cellular response.

In summary, our study demonstrated that an 810 nm diode laser with subthreshold power (fluence lower than 84.08 J/cm^2^) applied for 60 s can stimulate Hsp70 production in cultured choroid-retinal endothelial cells without obvious cell damage. The Hsp70 expression was found to peak at 12 to 18 h following laser exposure, and returned to baseline at about 48 h. These results may facilitate designing more effective laser therapies.
